# The revised zone of partial preservation (ZPP) in the 2019 International Standards for Neurological Classification of Spinal Cord Injury: ZPP applicability in incomplete injuries

**DOI:** 10.1038/s41393-023-00950-x

**Published:** 2024-01-08

**Authors:** Christian Schuld, EMSCI study group, Rainer Abel, Armin Curt, Yorck-Bernhard Kalke, Jiri Kriz, Doris Maier, Norbert Weidner, Steven Kirshblum, Keith Tansey, ASIA International Standards Committee, Randal Betz, Fin Biering-Sørensen, Stephen P. Burns, William Donovan, Daniel E. Graves, James Guest, Linda Jones, Andrej Krassioukov, Mary Jane Mulcahey, Mary Schmidt Read, Gianna M. Rodriguez, Kristen Walden, Rüdiger Rupp

**Affiliations:** 1 Spinal Cord Injury Center, Heidelberg University Hospital, Schlierbacher Landstr. 200a, 69118 Heidelberg, Germany; 2 Kessler Institute for Rehabilitation, West Orange, NJ USA; 3 Rutgers New Jersey Medical School, Newark, NJ USA; 4 Center for Neuroscience and Neurological Recovery, Methodist Rehabilitation Center, Jackson, MS USA; 5 Spinal Cord Injury Clinic, Jackson VA Medical Center, Jackson, MS USA; 6 Departments of Neurosurgery and Neurobiology and Anatomical Sciences, University of Mississippi Medical Center, Jackson, MS USA; 7 Spinal Cord Injury Center, Klinik Hohe Warte, Bayreuth, Germany; 8 Spinal Cord Injury Center, Balgrist University Hospital, Zurich, Switzerland; 9 RKU Universitäts- und Rehabilitationskliniken Ulm, Ulm, Germany; 10 Spinal Cord Unit, Department of Rehabilitation and Sports Medicine, 2nd Faculty of Medicine, Charles University and University Hospital Motol, Prague, Czech Republic; 11 BG Unfallklinik Murnau, Murnau, Germany; 12 Institute for Spine and Scoliosis, Ocean City, NJ USA; 13 Department for Spinal Cord Injuries, Rigshospitalet, University of Copenhagen, 575, Copenhagen, Denmark; 14 Department of Rehabilitation Medicine, University of Washington School of Medicine, 582, Seattle, WA USA; 15 Spinal Cord Injury Service, VA Puget Sound Health Care System, Seattle, WA USA; 16 Institute for Rehabilitation and Research, Houston, TX USA; 17 Thomas Jefferson University, Philadelphia, PA USA; 18 University of Miami, Miller School of Medicine, Miami, FL USA; 19 GF Strong Rehabilitation Center, Vancouver, BC Canada; 20 Vancouver Coastal Health Research Institute, Vancouver Coastal Health, Vancouver, BC Canada; 21 International Collaboration on Repair Discovery (ICORD), University of British Columbia, Vancouver, BC Canada; 22 Center for Outcomes and Measurement, Jefferson College of Rehabilitation Sciences, Thomas Jefferson University, Philadelphia, PA USA; 23 Magee Rehabilitation Hospital, Jefferson Health, Philadelphia, PA USA; 24 Michigan Medicine, Department of Physical Medicine and Rehabilitation, Ann Arbor, MI USA; 25 Praxis Spinal Cord Institute, Vancouver, BC Canada

**Keywords:** Outcomes research, Spinal cord diseases

## Abstract

**Study design:**

Consensus process.

**Objectives:**

To provide a reference for the Zone(s) of Partial Preservation (ZPP) in the 2019 International Standards for Neurological Classification of Spinal Cord Injury (ISNCSCI) and analyze the initial impact of applicability of the revised ZPPs. Revisions include the use of ZPPs in selected incomplete injuries (in addition to prior use in sensorimotor complete injuries). Specifically, the revised motor ZPPs are applicable bilaterally in injuries with absent voluntary anal contraction (VAC) and the revised sensory ZPP for a given side is applicable if deep anal pressure (DAP), light touch and pin prick sensation in S4-5 are absent on that side.

**Setting:**

Committee with 16 ISNCSCI experts and datasets from the European Multicenter Study about Spinal Cord Injury (EMSCI).

**Methods:**

Occurrence frequencies of applicable ZPPs were determined in an EMSCI cohort consisting of two ISNCSCI examinations from 665 individuals with traumatic SCI.

**Results:**

Motor ZPPs were derived in 35.2% of all datasets of incomplete injuries, while sensory ZPPs are much less frequent (1.0%). Motor ZPPs are applicable in all American Spinal Injury Association Impairment Scale (AIS) B datasets (mean ZPP length: 0.9 ± 1.0 segments), in 55.4% of all AIS C datasets (ZPP length: 11.8 ± 8.2 segments) and in 9.9% of the AIS D datasets (ZPP length: 15.4 ± 7.9 segments).

**Conclusions:**

The revised ZPP allows for determining motor ZPPs in approximately 1/3 of all incomplete injuries. The broadened applicability enables the use of ZPPs beyond complete injuries for complementary description of residual functions in more individuals.

**Sponsorship:**

N/A

## Introduction

The International Standards for Neurological Classification of Spinal Cord Injury (ISNCSCI) [1] represent the most established neurological assessment for characterizing the level and severity of a spinal cord injury (SCI). In addition to determining specific levels (motor, sensory and neurological) and the American Spinal Injury Association (ASIA) Impairment Scale (AIS) grade, the zone of partial preservation (ZPP) is another important aspect of the ISNCSCI. The ZPPs refer to “those dermatomes and myotomes caudal to the sensory and motor levels with partially preserved function” [1] and are documented as up to four distinct segments defined for right and left sides. ZPPs provide important information to clinicians and researchers, because they (1) represent – together with the total scores, levels and AIS – important variables for a quick characterization of the neurological impairments of a person with SCI; (2) support effective communication among clinicians; (3) are among the most important predictors of neurological recovery in individuals with complete (AIS A) traumatic SCI [2], and (4) might support the identification of different recovery patterns.

In the first ISNCSCI edition released in 1982 [3], ZPPs were applicable for all injuries (and were defined as segments with preserved function up to three levels below the neurological level of injury (NLI)) and used for distinction between neurological complete and incomplete injuries. If no motor and/or sensory function was preserved more than 3 segments below the NLI, the injury was classified as complete (based on the Frankel Scale [4]). With the 1992 revision [5], a major change in the definition of the completeness of an injury was introduced based on the presence or absence of functions in the lowest sacral segments only [6]. This had a major impact on the ZPPs which were from then on only defined in complete (AIS A) injuries [7]. Unfortunately, the application of ZPPs only in AIS A injuries limits their use for documentation of residual functions only to motor and sensory complete injuries. By applying ZPPs only in people with AIS grade A, important information might be lost in the classification for cases where only sensory **or** motor function is absent in the lowest sacral segments, but not sensory **and** motor function.

Therefore, a revised ZPP independent of the AIS was introduced in the current eighth edition of ISNCSCI [1]. The scope of the ZPP was changed so that the applicability of the ZPP becomes independent from the AIS classification and that the applicability is checked separately for all four ZPPs using this definition:

*“This term [ZPP], used only in injuries with absent motor (no VAC) OR sensory function (no DAP, no LT and no PP sensation) in the lowest sacral segment S4-5, refers to those dermatomes and myotomes caudal to the sensory and motor levels with partially preserved functions. The most caudal segment with some sensory and/or motor function defines the extent of the sensory or motor ZPP respectively and are documented as four distinct levels (R-sensory, L-sensory, R-motor, and L-motor).”*

*Abbreviations: Voluntary anal contraction (VAC), Deep anal pressure (DAP), Light touch (LT), Pin prick (PP)*

ZPP definitions in the International Standards Training E Program (InSTeP) e-learning tool [8] and worksheet [9] were changed accordingly.

The International Standards Committee of the ASIA with members of the International Spinal Cord Society (ISCoS) serves as the custodian of ISNCSCI and is committed to revise ISNCSCI transparently and based on evidence [10]. The aim of this work was (1) to provide the frequency of pertinent occurrence in a large cohort of people with SCI, especially in persons with incomplete injuries and (2) to offer discussion points to anticipated questions about the revised ZPPs.

This paper is intended to supplement the ISNCSCI booklet [1] and acts as a reference to support clinicians in the correct application of the revised ZPP definition.

## Methods

A representative [11] benchmark dataset from the European Multicenter Study about Spinal Cord Injury (EMSCI) database consisting of ISNCSCI datasets from 665 individuals (inclusion criteria: traumatic SCI, age ≥ 16 years) was used. For each individual, early and late ISNCSCI datasets were included: the first assessed within the first 30 days after injury and the second assessed approximately 12 months after injury. These two time points were chosen to allow comparison with other databases [12] and to form a representative cohort of people for better estimation of ZPP incidence.

First, ZPPs of all ISNCSCI datasets were recalculated using the ZPP rules of the 2019 ISNCSCI revision. The percentage of datasets of incomplete injuries, in which ZPPs are applicable, was determined. Additionally, on each side of the body the ZPP length given as number of segments between the sensory/motor levels and the corresponding most caudal segment with any function (caudal extent of the ZPP) was descriptively analyzed. For example, in a case with a right sensory level of T6 and the most caudal segment on the right with preserved sensory function of T10, which is recorded as right sensory ZPP level, the ZPP length would be 4 segments.

The revised ZPP was implemented into the EMSCI ISNCSCI calculator [13] to recalculate all ISNCSCI records. Supplemental Material 1 outlines the technical implementation of the function ZPP_2019 in pseudo code as reference for ISNCSCI computer algorithm developers.

The occurrence frequencies and lengths of the ZPPs in all datasets of incomplete injuries were analyzed separately for motor and sensory ZPPs and grouped by body side and AIS grades.

## Results

In the benchmark dataset consisting of 1330 ISNCSCI assessments in the early and late stage after SCI from 665 individuals (age at injury: 43.1 ± 17.8 years, 135 (20.3%) female), the age distribution grouped by increments of 15 years [14], was as follows: 30.8% 16–30 years, 24.4% 31–45 years, 23.8% 46–60 years, 18.3% 61–75 years and 2.7% 76+ years.

The early ISNCSCI assessment was captured 11.8 ± 7.6 days after injury whereas the late ISNCSCI examination was obtained nearly one year after injury (357.1 ± 53.7 days after injury). AIS and AIS-grouped NLI range [14] distributions separated by the time point of assessment are shown in Table 1. In the initial assessment, approximately half (46%) of the included individuals had complete injuries and 35% had a cervical NLI.

**Table 1:** Distribution of the American Spinal Injury Association Impairment Scale (AIS) and the Neurological Level of Injury (NLI) for the early (11.8 ± 7.6 days after injury) and the late (357.1 ± 53.7 days after injury) time window for all 665 included individuals.

Acute assessment				Chronic assessment			
AIS	AIS %	NLI	NLI %	AIS	AIS %	NLI	NLI %
A	46.0	C1–C4	11.6	A	34.9	C1–C4	6.9
		C5–C8	7.2			C5–C8	4.4
		T1–S5	27.2			T1–S5	23.6
B	11.3	C1–C4	3.2	B	8.4	C1–C4	1.4
		C5–C8	3.0			C5–C8	3.5
		T1–S5	5.1			T1–S5	3.6
C	17.5	C1–C4	7.1	C	8.9	C1–C4	1.5
		C5–C8	3.3			C5–C8	2.3
		T1–S5	7.1			T1–S5	5.1
D	25.3	all levels		D	46.3	all levels	
E	0.0	all levels		E	1.5	all levels	

To check for correctness of the revised ZPP computer implementation, ZPPs for AIS grade A were calculated with both the validated and the revised ZPP algorithm. No differences in the calculated ZPPs between the old and the new ZPP algorithm and implementation were found as expected.

### Motor ZPPs

**Fig. 1:**
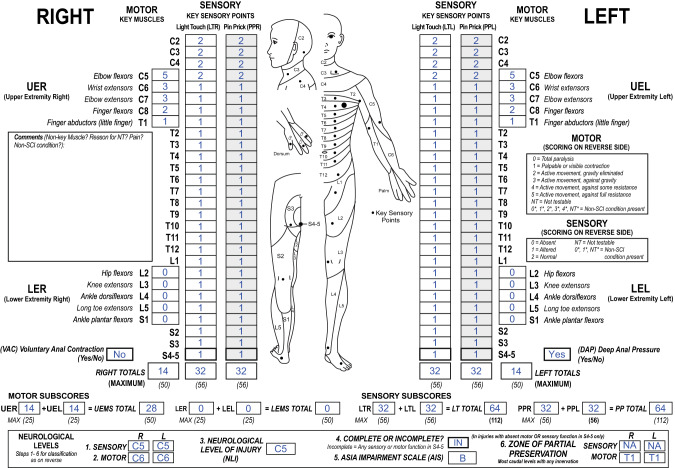
International Standards for Neurological Classification of Spinal Cord Injury (ISNCSCI) example illustrating the revised motor zones of partial preservation (ZPP) in a sensory incomplete injury. According to the 2019 ISNCSCI revision, motor ZPPs are applicable and should be documented on both sides of the body in all cases with absent voluntary anal contraction (VAC) including patients with incomplete injuries. This case is classified as neurological level of injury C5 and American Spinal Injury Association Impairment Scale grade B. As VAC is absent, motor ZPPs of T1 on both sides indicating substantial motor function preserved in both upper extremities below the motor level (C6) down to T1 are recorded as right and left motor ZPP.

In the 2019 ISNCSCI revision, motor ZPPs are applicable in all cases with absent VAC (prototypical case is illustrated in Fig. 1). As a result, motor ZPPs can be determined in all AIS B injuries, because an injury with present VAC is classified as motor incomplete (AIS grade C or grade D).

However, the length of the motor ZPPs in individuals classified as AIS B will not exceed three segments, because by definition, an injury with sparing of sensory function in the lowest sacral segments S4-5 and of motor function more than three levels below the ipsilateral motor level results in a motor incomplete status and therefore an AIS C or D classification. In contrast, AIS A injuries can have a length of the motor ZPP exceeding three segments.

Among the 131 pooled ISNCSCI datasets with AIS B classification (Table 2), the length of the right and left motor ZPPs are as follows: 0 segments (right 61 datasets, left 60 datasets), 1 segment (right 29, left 38), 2 segments (right 30, left 24), 3 segments (right 11, left 9). The median (25–75% percentile) ZPP length for both sides is 1 (0–2) segments.

**Table 2:** Occurrence frequencies grouped by AIS grade of motor (A) and sensory (B) zones of partial preservation (ZPPs) and their length in number of spinal segments below the corresponding motor/sensory levels.

A: Motor ZPPs in datasets with absent VAC								
			Number (percentage) of motor ZPPs		Motor ZPP length in spinal segments below motor level			
AIS	Assessment time point	*N*	Right	Left	Right		Left	
					Median (25–75% percentile)	Mean ± SD	Median (25–75% percentile)	Mean ± SD^b^
A	Pooled	538	538 (100%)	538 (100%)	0 (0–1)	1.29 ± 3.41	0 (0–1)	1.28 ± 3.28
	Early	306	306 (100%)	306 (100%)	0 (0–1)	1.51 ± 3.86	0 (0–0.75)	1.44 ± 3.60
	Late	232	232 (100%)	232 (100%)	0 (0–1)	0.99 ± 2.70	0 (0–1)	1.07 ± 2.80
B	Pooled	131	131 (100%)	131 (100%)	1 (0–2)	0.93 ± 1.02	1 (0–1.5)	0.86 ± 0.95
	Early	75	75 (100%)	75 (100%)	1 (0–2)	0.91 ± 1.02	1 (0–2)	0.93 ± 0.95
	Late	56	56 (100%)	56 (100%)	1 (0–2)	0.96 ± 1.03	0 (0–1)	0.77 ± 0.95
C	Pooled	175	97 (55.4%)	97 (55.4%)	8 (4–20)	11.14 ± 8.45	14 (5–20)	12.54 ± 7.86
	Early	116	60 (51.7%)	60 (51.7%)	6 (2–20.25)	10.53 ± 8.85	17.5 (5–20.25)	13.53 ± 7.86
	Late	59	37 (62.7%)	37 (62.7%)	12 (4–20)	12.14 ± 7.77	9 (4–19)	10.92 ± 7.69
D	Pooled	476	47 (9.9%)	46^a^ (9.7%)	20 (6–21)	14.81 ± 8.54	19.5 (8.25–21)	16.04 ± 7.14
	Early	168	21 (12.5%)	21 (12.5%)	20 (6–22)	14.86 ± 8.67	20 (9–21)	16.62 ± 6.91
	Late	308	25 (8.1%)	26 (8.4%)	19 (6–20.75)	14.77 ± 8.61	19 (6–20.75)	15.56 ± 7.44
E	Pooled	10	0 (0%)	0 (0%)	–		–	
	Early	0	0 (0%)	0 (0%)	–		–	
	Late	10	0 (0%)	0 (0%)	–		–	

B: Sensory ZPPs in datasets with absent DAP and absent LT/PP sensation in S4-5								
			Number (percentage) of sensory ZPPs		Sensory ZPP length in spinal segments below sensory level			
AIS	Assessment time point	*N*	Right	Left	Right		Left	
					Median (25–75% percentile)	Mean ± SD^b^	Median (25–75% percentile)	Mean ± SD^b^
A	Pooled	538	538 (100%)	538 (100%)	2 (1–5)	3.51 ± 4.26	2 (1–5)	3.54 ± 4.33
	Early	306	306 (100%)	306 (100%)	2 (1–5)	3.70 ± 4.67	2 (1–5.75)	3.70 ± 4.76
	Late	232	232 (100%)	232 (100%)	0 (0–1)	0.99 ± 2.70	0 (0–1)	1.07 ± 2.80
B	Pooled	131	2 (1.5%)	0 (0%)	4.5 (2.75–6.25)	–	–	–
	Early	75	0 (0%)	0 (0%)	–	–	–	–
	Late	56	2 (3.6%)	0 (0%)	4.5 (2.75–6.25)	–	–	–
C	Pooled	175	6 (3.4%)	7 (4.0%)	3 (1–6.5)	–	5 (2–8)	–
	Early	116	4 (3.4%)	4 (3.4%)	6 (4–10.75)	–	6 (4–10.75)	–
	Late	59	2 (1.7%)	3 (5.1%)	0.5 (0.25–0.75)	–	3 (1.5–6)	–
D	Pooled	476	0 (0%)	0 (0%)	–	–	–	–
	Early	168	0 (0%)	0 (0%)	–	–	–	–
	Late	308	0 (0%)	0 (0%)	–	–	–	–
E	Pooled	10	0 (0%)	0 (0%)	–	–	–	–
	Early	0	0 (0%)	0 (0%)	–	–	–	–
	Late	10	0 (0%)	0 (0%)	–		–	–

For this analysis, all 1330 ISNCSCI datasets of the 665 included individuals were analyzed individually for the early and the late assessment time points, and on a group level including all assessments from both time points (rows “pooled”).

*AIS* American Spinal Injury Association Impairment Scale, *DAP* deep anal pressure, *LT* light touch, *PP* pin prick, *VAC* voluntary anal contraction.

^a^In one AIS D case, the motor level is not determinable on the left body side and therefore the extent of the ZPP cannot be calculated, which explains the difference between left and right sample sizes.

^b^Mean and standard deviation are only calculated for at least 10 samples.

In datasets of motor incomplete injuries (AIS grade C (*n* = 175) or grade D (*n* = 476)), motor ZPPs are only applicable when VAC is absent. These datasets with absent VAC were classified as AIS grade C or grade D due to sparing of motor function more than three levels below the ipsilateral motor level on at least one side of the body. The distribution of the ZPP lengths is more diverse in datasets classified as AIS grade C or grade D than in datasets classified as AIS grade B ranging from 0 to the maximum of 25 segments (sparing of motor function until S1 in a case with a motor level of C1) with peaks at 4 segments (borderline cases between AIS grade B and AIS grade C/D) and 21 segments (incomplete C4/C5 injuries with sparing of motor function to L5/S1).

Table 2 depicts occurrence frequencies and average ZPP lengths grouped by AIS grade for motor and sensory ZPPs. In 55.4% (97/175) of the pooled AIS C datasets, VAC was absent with a median motor ZPP length of 8 (4–20) (right) and 14 (5–20) (left) segments. Of the pooled AIS D datasets, 9.9% (47/476) had absent VAC with median motor ZPP lengths of 20 (6–22) (right) and 19.5 (8.25–21) (left) segments. Overall, motor ZPPs can be derived in 35.2% (131 + 97 + 47)/(131 + 175 + 476 = 782) of the datasets of all incomplete injuries (early: 43.5%, late: 28.0%). Table 2 also contains occurrence frequencies and ZPP lengths for the early and late assessment time points. Supplemental material 2 depicts the ZPP length distributions as histograms.

### Sensory ZPPs

**Fig. 2:**
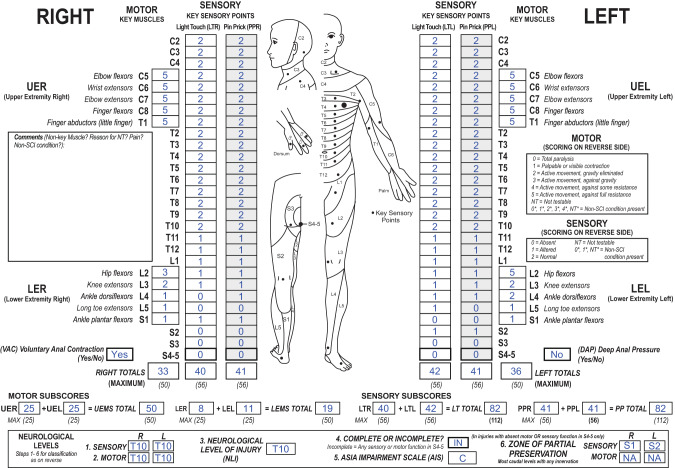
International Standards for Neurological Classification of Spinal Cord Injury (ISNCSCI) example illustrating residual sensory function below the sensory level in a motor incomplete injury with absent sensory function in S4-5. According to the 2019 ISNCSCI revision, the sensory zone of partial preservation (ZPP) on a given side is applicable in the absence of sensory function in S4-5 (light touch (LT), pin prick (PP)) on this side and absent deep anal pressure (DAP). In cases with present DAP, sensory ZPPs on both sides are not applicable (and noted as “not applicable (NA)”). In a case with absent DAP, but LT or PP sensation preserved in S4-5 on one side, the sensory ZPP on this side is not applicable (and should be noted as ‘NA’). This case is classified as American Spinal Injury Association Impairment Scale grade C (presence of VAC and less than half of the key muscles below the neurological level of injury (NLI) with a motor score ≥ 3) with an NLI of T10. Because there is no sensory function preserved in S4-5, sensory ZPPs (right: S1, left: S2) can be determined to document the substantial residual sparing (right: 8 segments, left: 9 segments) of sensory functions caudal to the sensory levels.

In the 2019 ISNCSCI revision, sensory ZPPs on a given side of the body can be provided in all cases with absent DAP and LT and PP sensation in S4-5 on that side. In the sample of datasets of incomplete injuries (*n* = 782), sensory ZPPs (prototypical case is depicted in Fig. 2) are much less frequent than motor ZPPs: 1.0% (8/782) right ZPPs and 0.9% (7/782) left ZPPs (Table 2).

**Fig. 3:**
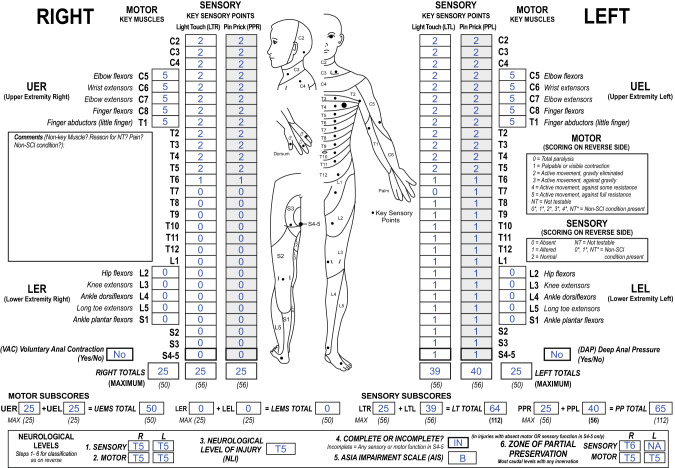
International Standards for Neurological Classification of Spinal Cord Injury (ISNCSCI) example of a rare case with a unilateral sensory zone of partial preservation (ZPP). This worksheet shows the examination results of  a person with a sensory incomplete (American Spinal Injury Association Impairment Scale grade B) injury, but absent deep anal pressure as well as missing light touch and pin prick sensation on one side of the body.

Five AIS C datasets from different individuals (4 early; 1 late), in which VAC is present, but DAP is absent as well as LT and PP on both body sides in the lowest sacral segments S4-5, had bilateral sensory ZPPs. Five unilateral sensory ZPPs (5/1330 = 0.4% of the whole cohort, 5/782 = 0.6% of all incomplete injuries) were found (3 on the right body side, 2 on the left side). Two of these datasets were AIS B and three were AIS C injuries. These datasets with a unilateral sensory ZPP are characterized by absent DAP and absent LT and PP sensation in S4-5 on one side of the body and preserved LT or PP sensation in S4-5 on the other side. Figure 3 depicts one of these datasets with a sensory incomplete injury classified as AIS grade B due to the preservation of LT and PP sensation in S4-5 on the left side. DAP is absent as well as the right LT and PP sensation in S4-5. Therefore, a right sensory ZPP is applicable and can be determined as T6.

## Discussion

While levels and severity (AIS) are basically applicable in all injuries, the ZPPs are not. In most of the incomplete injuries, sensorimotor functions extend to the lowest sacral segments. Therefore, in previous ISNCSCI revisions, ZPPs were only applicable in complete (AIS grade A) injuries defined by the total absence of sensory and motor functions in the lowest sacral segments [15]. However, the ASIA International Standards Committee agreed that this scope had limited face validity: (1) It did not match the concept of providing spinal segmental levels separately for each assessment modality, namely the sensory and motor levels, and (2) it did not allow the use of ZPPs for clinical characterization of patients with incomplete injuries where only motor or sensory function is absent in the lowest sacral segments. With the current 2019 ISNCSCI revision, the scope of the ZPPs was redefined so that the applicability of motor and sensory ZPPs is checked separately.

The committee considers the basic definition of the ZPP, which “refers to those dermatomes and myotomes caudal to the sensory and motor levels that remain partially innervated” [15, 16] to have high face validity. Therefore, neither this basic ZPP definition nor the wording was changed for the 2019 ISNCSCI revision. The committee explicitly wanted to emphasize that the ZPP starts rostrally at the sensory/motor level and ends at the most caudal segment with preserved sensory/motor function on that side [10]. This means that four independent zones of partial preservation, the right and left motor ZPPs as well as the right and left sensory ZPPs, together with their lengths are defined. In respect to clinical meaningfulness, however, it makes sense to restrict the application of the ZPP to those situations where the caudal extent of that ZPP does not reach to the lowest sacral segments. In the previous ISNCSCI revision [15], all four ZPPs were defined to be only applicable in AIS A injuries with fully absent motor (VAC) and sensory (LT and PP sensation in S4-5, DAP) functions in the lowest sacral segments [15].

The recording of motor ZPPs in cases with absent VAC is of substantial clinical value, because they provide complementary information to the Upper and Lower Extremity Motor Scores regarding the extent of the preserved motor functions below the motor level.

With the revised ZPP, motor ZPPs are applicable in approximately 1/3 of all datasets with incomplete injuries in a benchmark dataset of individuals with traumatic SCI queried from the EMSCI database. We found a slightly higher applicability in the early phase (43.5%) as compared to late phase after SCI (28%) due to the recovery of voluntary anal contraction over time.

In incomplete injuries, motor ZPPs are much more frequent than sensory ZPPs. A sensory ZPP is only defined in incomplete injuries with present VAC, but no sensory function in the lowest sacral segments on a given side of the body. This constellation was found to be rather rare in the analyzed EMSCI dataset, but is in line with other data sources, e.g. the Spinal Cord Injury Model Systems (SCIMS) database (e.g. Table 3 of [12]). A SCIMS analysis of the initial AIS grades reveals that present VAC and absence of any sensation in S4-5 (LT, PP and DAP) is found in 3.2% (EMSCI: 3.4%) of all AIS C patients and in 0.1% (EMSCI: 0%) of all AIS D patients. Sparing of sensory, but not motor (VAC) function in the lowest sacral segments is found in 40.6% (EMSCI: 51.7%) of all AIS C patients and in 12% (EMSCI: 12.5%) of all AIS D patients. These numbers obtained independently in two large, representative cohorts of individuals with SCI underline the rationale and usefulness of the refined ZPP scope with which motor ZPPs can be reported in all AIS B injuries, approx. 50% of all AIS C and approx. 10% of all AIS D injuries.

An important question is the significance of the revised ZPP definition for prediction of neurological recovery. This question will be analyzed in detail in a follow-up publication. It is anticipated that prediction models using ZPP variables from within the first week after injury will even more accurately predict the outcome 1 year after injury than from the average assessment time point of 11.8 ± 7.6 days after injury applied in this study. A recent review [17] concludes that examinations conducted within the first 24 h to one week after injury are highly reliable and predictive of the late neurological outcome.

This revision implies some noteworthy features and characteristics, which are discussed in the following ”frequently asked question”-alike subchapters.

### Is the revised ZPP compatible with previous revisions?

The ASIA International Standards Committee placed a strong focus on backward compatibility. In particular, the revised ZPP definition is compatible in patients with complete SCI, for which all ZPPs determined according to former revisions are identical to ZPPs determined with the 2019 revision. However, with the 2019 revision, more cases will have applicable ZPPs, predominantly motor ZPPs.

This feature will help to compare populations of existing studies with those of future studies. In future studies, authors are strongly advised to report the ZPPs distributions grouped by AIS. The ZPP distribution of the AIS A group equals to the distribution of the ZPPs according to the 2015 ISNCSCI update [15].

### Can a sensory ZPP be present only on one side of the body?

While motor ZPPs are always applicable bilaterally in cases with absent VAC, the revised ZPP definition implies the possibility of a unilateral sensory ZPP. A unilateral sensory ZPP occurs, when DAP is absent, but sensory function in S4-5 is preserved only on one side of the body. Figure 3 exemplarily depicts such a case with absent DAP and a unilateral sensory ZPP of T6 on the right side, but preserved sensory function in S4-5 on the left and therefore a non-applicable left sensory ZPP. However, such a constellation occurs very rarely, which is supported by our analysis of the EMSCI datasets with only 5 of 1330 ISNCSCI datasets having a unilateral sensory ZPP.

### Are non-key muscle functions incorporated in the determination of ZPPs?

The ASIA International Standards Committee agreed many years ago that preserved functions of non-key muscles are NOT relevant for AIS classification except for the differentiation between AIS grade B and grades C/D: In an individual with an apparent AIS B classification, non-key muscle functions more than 3 levels below the motor level on each side should be tested to most accurately classify the injury, i.e. to differentiate between AIS grade B and grades C/D. In ISNCSCI instructional courses [18], it is typically trained to ask sensory incomplete but motor complete patients as last step in the motor examination: “Can you move anything else [below the motor level], which I have not tested yet?”

**Fig. 4:**
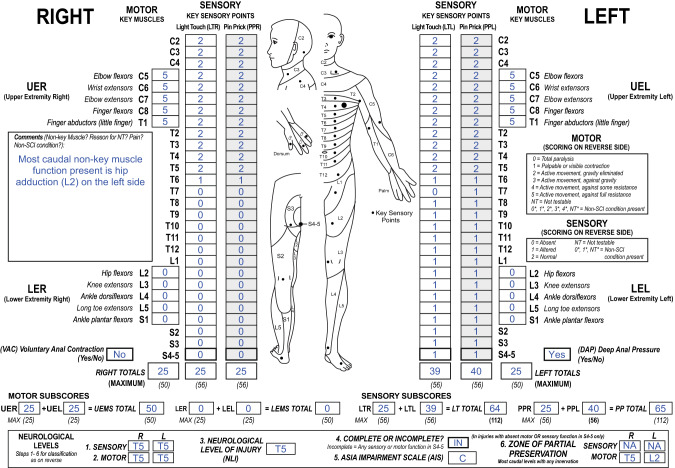
International Standards for Neurological Classification of Spinal Cord Injury (ISNCSCI) case with the motor zone of partial preservation (ZPP) determined by a preserved non-key muscle function. The left motor level in this case is T5 with preserved left hip adduction associated to the spinal segment L2. Due to the preservation of sensory function in the lowest sacral segment S4-5, preserved motor function more than three segments below the motor level, but absent lower extremity motor functions, this individual is classified as AIS grade C. Because of the impact of the preserved non-key muscle function in the left L2 segment on the American Spinal Injury Association Impairment Scale classification, L2 is recorded as left motor ZPP.

ISNCSCI worksheet (backside) and booklet (page 35) [16] contain a reference table of important muscle functions with an assignment to a corresponding spinal root level. A case, which would be classified as AIS grade B based on the examination results of the key muscles only, is classified as motor incomplete and therefore AIS grade C, if the most caudal root level of preserved non-key muscle functions is located more than three segments below the ipsilateral motor level. Figure 4 depicts such a case, which is classified as AIS grade C due to a sensory and motor level at T6 and preserved hip adduction associated with segment L2 on the left side. As this non-key muscle function more than 3 segments below the motor level is decisive for the AIS C classification, the corresponding caudal extent of the left motor ZPP is also set to L2.

### When to use ‘not applicable (NA)’ or ‘not determinable (ND)’?

The ZPP boxes in the right lower corner of the worksheet should always be completed. Empty boxes should be avoided to clearly indicate that the examiner did not simply forget to fill in the box. The committee decided that NA (‘not applicable’) should be recorded, when a particular ZPP is not applicable versus leaving the box blank.

Motor ZPPs are not applicable, if VAC is present. Sensory ZPPs are not applicable, if DAP is present. If DAP is absent, a sensory ZPP is not applicable when LT or PP sensation is preserved (scored one or two) in S4-5 on the respective body side.

In other words, the sensory / motor ZPPs are only applicable when sensory / motor function is absent in the lowest sacral segments. Anecdotally, beginners of ISNCSCI often record S4-5 as sensory ZPPs in incomplete injuries (unpublished results from the ISNCSCI instructional courses conducted in the EMSCI network [19]). Although, S4-5 and NA basically convey the same information, the recording of NA is recommended to clearly indicate that function is preserved at the lowest sacral segments and there is no absent function caudal to the ZPP.

ZPPs might be not determinable (ND), if the ISNCSCI dataset contains not testable (NT) score(s) [13]. For example, a motor ZPP is not determinable, if a motor score below the motor level is NT and motor function is totally absent in all key muscles caudal to this NT myotome. In this situation, the motor ZPP is not uniquely determinable as it depends on the “real” motor score of the not testable myotome. A motor score graded 1 or better would lead to a motor ZPP at that segment. A motor score of 0 would result in a more rostral motor ZPP. As the motor ZPP starts at the motor level and ends at the most caudal segment with preserved motor function, the motor level has to be considered in the decision whether a motor ZPP is defined or not. If the motor level is ND and no motor functions are preserved caudal to the not-determinable motor level, the motor ZPP equals the motor level and is therefore also not determinable.

Please see Schuld et al. [13] for a comprehensive discussion of this determinability problem not only for ZPPs but for all ISNCSCI classification variables.

Not testable (NT) is only used for grading of the sensory and motor examination (including DAP and VAC), and does not apply to any classification variable like the levels, the AIS or the ZPPs.

### When to tag ZPPs with an asterisk?

The 2019 ISNCSCI revision introduced a taxonomy for documentation of non-SCI conditions which are impacting the examination results and the classification. There might be cases, when ZPPs need to be tagged with an asterisk to indicate that they are based on clinical assumptions. More details can be found in [20].

## Conclusion

In the 2019 ISNCSCI revision, the scope of the applicability of ZPPs was broadened to include not only complete but also certain incomplete injuries. Motor ZPPs are applicable bilaterally in all cases with absent voluntary anal contraction. A sensory ZPP for a given side is applicable, if deep anal pressure sensation is absent and both light touch and pin prick sensation in S4-5 are absent on that side. An analysis of ISNCSCI datasets from a representative EMSCI cohort showed that the revised ZPP definition does not substantially change the overall occurrence frequency of sensory ZPPs. However, it allows the determination of motor ZPPs in approximately one third of all incomplete injuries. This will foster the use of ZPPs in research (e.g. for prognosis of at-level recovery) and for communication among clinicians as well as patients as it helps - together with the levels and the AIS - to summarize the neurological characteristics of a SCI.

### Supplementary information

Supplemental Material 1

Supplement Material 2

## Data Availability

The EMSCI datasets are not publically available. Upon reasonable request the EMSCI scientific board might initiate a collaboration to share data for a specific scientific question.
