# Supplementary material for: The revised zone of partial preservation (ZPP) in the 2019 International Standards for Neurological Classification of Spinal Cord Injury: ZPP applicability in incomplete injuries
Source: Spinal Cord. 2024 Jan 8;62(2):79–87. doi: 10.1038/s41393-023-00950-x (PMC10853064; doi:10.1038/s41393-023-00950-x)
Supplement: Supplementary file 1 — Supplemental Material 1 [file 41393_2023_950_MOESM1_ESM.docx]

# Supplemental Material 1

## Technical implementation of the Zone of Partial Preservation (ZPP) as reference for computer algorithm developers

The process for determination of the Zone of Partial Preservation (ZPP) variables are implemented as follows in the EMSCI ISNCSCI calculator (https://ais.emsci.org) [1]:

1. For determination of a sensory ZPP of a given side, copy LT and PP scores of all segments on this side into a temporary variable “scores” as

scores[“Sensory”, side, seg] = determinability(LT[side, seg], PP[side, seg]);

side ϵ (Right, Left)
seg ϵ (C1, C2, C3, …, S1, S2, S3, S4-5)

The determinability function transfers LT[seg] and PP[seg] of a given side into a single segmental sensory score in the following way (see footnote):

1. LT[seg] = PP[seg] = 0
   🡺 determinability(LT[seg], PP[seg]) = 0
2. (LT[seg] = NT and PP[seg] = 0) or (LT[seg] = 0 and PP[seg] = NT)
   🡺 determinability(LT[seg], PP[seg]) = NT
3. all other combinations of LT[seg] and PP[seg]
   🡺 determinability (LT[seg], PP[seg]) = 1
   1. If DAP is present, set LT[Right, S4-5] = 1 and LT[Left, S4-5] = 1. This accounts for the applicability of sensory ZPPs only in cases with absent DAP.
4. For determination of a motor ZPP of a given side, copy motor scores MS[side, seg] of all key muscles of this side into the same temporary variables “scores” as:

scores[“Motor”, side, seg] = determinability(MS[side, seg]); seg ϵ (C5, C6, .., T1, L2, L3,.., S1)
scores[“Motor”, side, seg] = 0 ; seg ϵ (C1, C2, C4, T2, T3,.., L1, S2, S3,
S4-5)

- 1. If VAC is present, set MS[Right, S4-5] = 1 and MS[Left, S4-5] = 1. This accounts for the applicability of motor ZPPs only in cases with absent VAC.
  2. If the ASIA Impairment Scale is AIS grade B, set MS[side, non-key muscle root level]= 1 for the most caudal non-key muscle with preserved function on a given side.

1. Determine the most caudal segment with preserved function as ZPP[modality, side] for all four temporary variables scores[modality, side, seg] with modality ϵ (Sensory, Motor) and side ϵ (Right, Left) from 1. and 2. as follows:
   1. Starting at the lowest sacral segment, move segment-wise rostrally until the first segment with preserved function or NT is found, i.e. scores[modality, side, seg] ≠ 0.

3.2 If the scores value in this segment is NT, then the ZPP is ND.

3.3 For motor ZPPs only: If the found motor segment is equal or caudal to the corresponding motor level, record this segment as sensory/motor ZPP otherwise the corresponding level is used as ZPP. This ensures that the motor ZPP is not rostral to the motor level. This situation occurs if the motor level followed the sensory level.

1. If the result is S4-5, the ZPP is not applicable and denoted as “NA”.
2. The ZPP length in number of segments is calculated as ZPP segment – corresponding level:
   ZPP_length(modality, side) = ZPP(modality, side) - level(modality, side); modality ϵ (Sensory, Motor) and side ϵ (Right, Left)

* Not-testable sensory or motor scores represent a substantial challenge in ISNCSCI classification. A classification variable is only uniquely determinable, if all possible grades of the not-testable sensory or motor scores and their combinations lead to the same result [1]. To formalize the determination process of a classification variable in the presence of not-testable scores, a determinability function has been introduced. In case of sensory ZPPs, this function takes LT and PP scores of the most caudal segment with either preserved sensation (LT or PP scores of 1 or 2) or not testable sensory function (NT) into account. The sensory ZPP is not determinable, if both LT and PP are scored as not-testable. The determinability function returns NT in this case. If only LT or PP sensation is scored as not-testable, the determinability depends on the grading of the testable PP or LT sensation: If any sensory function is preserved (LT or PP score of 1 or 2) in the testable modality, the sensory ZPP is determinable (the determinability function returns 1 for further processing). If no sensory function is preserved in the testable modality (LT or PP score of 0), the sensory ZPP is not determinable (and NT is returned). The reason for this is that the not-testable sensory function could be either absent (scored as 0) or preserved (scored as 1 or 2), which leads to a not-uniquely determinable sensory ZPP.

[1] Schuld, C. *et al.* Computer implementation of the international standards for neurological classification of spinal cord injury for consistent and efficient derivation of its subscores including handling of data from not testable segments. *J. Neurotrauma* 29, 453-461 (2012).

Abbreviations: European Multicenter Study about Spinal Cord Injury (EMSCI), International Standards for Neurological Classification of Spinal Cord Injury (ISNCSCI), Deep anal pressure (DAP), Light touch (LT), Pin prick (PP), Motor score (MS), Voluntary anal contraction (VAC), Not testable (NT), American Spinal Injury Association (ASIA)
