# Supplementary material for: The revised zone of partial preservation (ZPP) in the 2019 International Standards for Neurological Classification of Spinal Cord Injury: ZPP applicability in incomplete injuries
Source: Spinal Cord. 2024 Jan 8;62(2):79–87. doi: 10.1038/s41393-023-00950-x (PMC10853064; doi:10.1038/s41393-023-00950-x)
Supplement: Supplementary file 2 — Supplement Material 2 [file 41393_2023_950_MOESM2_ESM.docx]

**Supplemental Material 2**


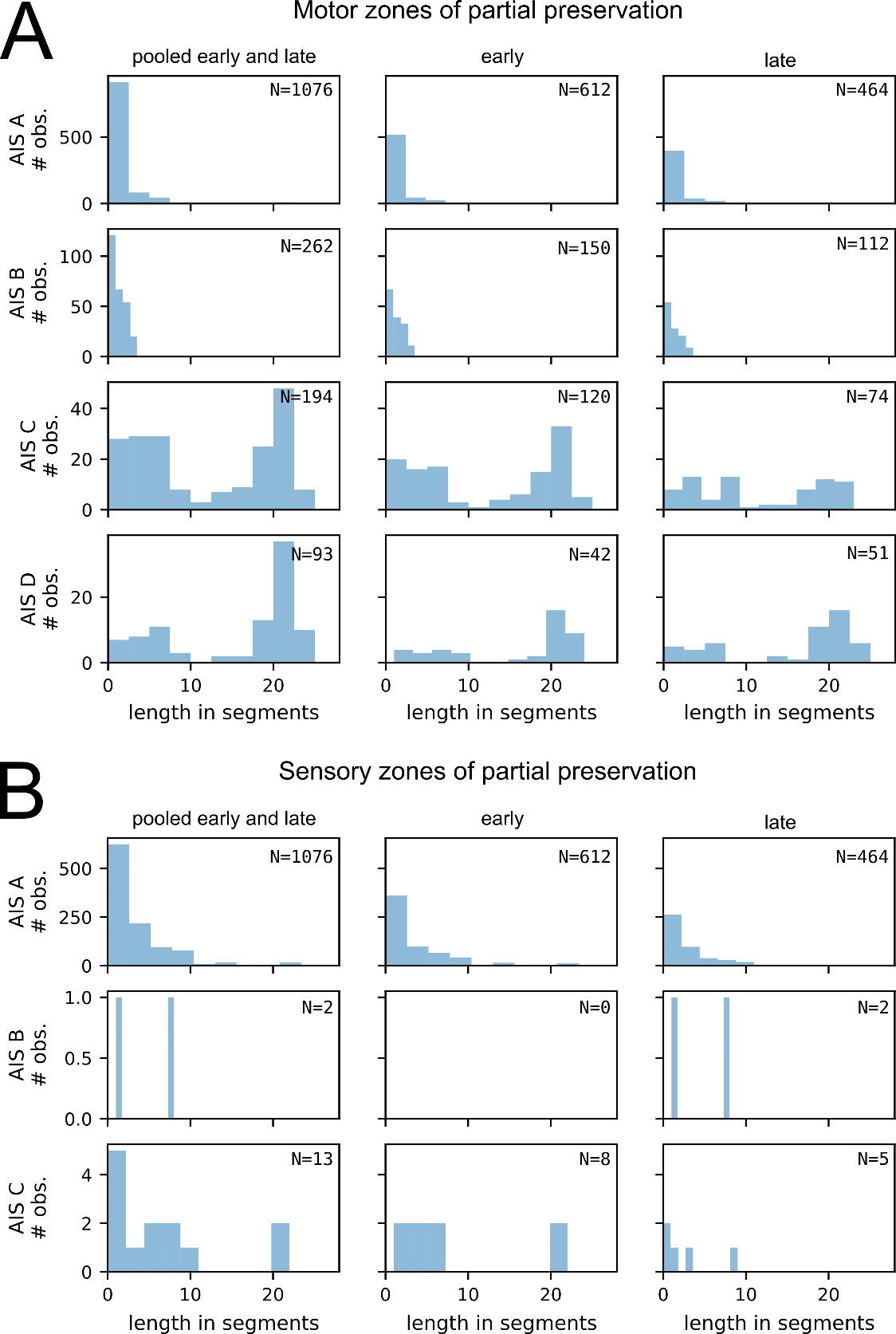


Subfigures A and B depict the distribution of the length of motor (subfigure A) and sensory (subfigure B) zones of partial preservation (ZPP), i.e. the number of spinal segments between the most rostral (of left and right body side) motor/sensory level and the most caudal segment with preserved motor/sensory function (ZPP).

Subfigures are arranged as follows: In rows, injury severity classified by the American Spinal Injury Association (ASIA) Impairment Scale (AIS); in columns, assessment time points (early: mean 11.8 ± 7.6 (standard deviation) days after injury; late: 357.1 ± 53.7 days after injury). The number of observations (# obs.) refers to the number of ZPPs, i.e. two observations (one for each bodyside) per ISNCSCI assessment. Motor incomplete injuries (AIS grade C or grade D) have a bimodal ZPP length distribution.
